# Coverage-dependent stability of Ru_*x*_Si_*y*_ on Ru(0001): a comparative DFT and XPS study

**DOI:** 10.1039/d4cp04069d

**Published:** 2024-11-16

**Authors:** Jonathon Cottom, Stefan van Vliet, Jörg Meyer, Roland Bliem, Emilia Olsson

**Affiliations:** a Advanced Research Center for Nanolithography Science Park 106 Amsterdam 1098 XG The Netherlands; b Institute of Theoretical Physics, Institute of Physics, University of Amsterdam Science Park 904 Amsterdam 1098 XH The Netherlands k.i.e.olsson@uva.nl; c Leiden Institute of Chemistry, Gorlaeus Laboratories, Leiden University 2300 RA Leiden The Netherlands; d van der Waals-Zeeman Institute, Institute of Physics, University of Amsterdam Science Park 904 Amsterdam 1098 XH The Netherlands

## Abstract

This work investigates the interaction of silicon with ruthenium, extending from Si-defect centers in ruthenium bulk to the adsorption of Si on the Ru(0001) surface. Using density functional theory (DFT) we calculate the interaction energies of up to 2 monolayers (MLs) of Si with this surface, uncovering the initial formation of ruthenium silicide (Ru_*x*_Si_*y*_). Our results demonstrate that Si readily forms substitutional defects (Si_Ru_) in bulk ruthenium. These defects are further stabilized on the Ru(0001) surface, resulting in a distinct propensity for forming Ru–Si_Ru_ mixed layers – which can thus be described by stoichiometry Ru_*x*_Si_*y*_. Overlayers of surface-adsorbed Si adatoms and Ru_*x*_Si_*y*_ mixed layers are iso-energetic at 0.5 ML, with the latter becoming increasingly energetically favored at higher Si coverages. We further examine the influence of Ru_*x*_Si_*y*_ formation with respect to oxide formation, focusing on coverage-dependent energy differences. Our results show Ru_*x*_Si_*y*_ layers are energetically favored with respect to the forming oxide for silicon and oxygen coverages above 1.1 ML, respectively. In addition, the formation of Ru_*x*_Si_*y*_ and the subsequent oxidation of Ru and Ru_*x*_Si_*y*_ were also investigated experimentally using *in situ* XPS. This confirmed the DFT prediction, with negligible oxide formation on the Ru_*x*_Si_*y*_ sample, whereas the unprotected Ru surface showed extensive RuO_2_ formation under the same conditions. Our study not only enhances the understanding of Ru surface chemistry but also suggests a straightforward computational approach for screening the oxidation resistance of surface coatings.

## Abstract 2

This work investigates the coverage dependent stability of silicon and oxygen on ruthenium, extending from defect centers in ruthenium bulk to the adsorption on the Ru(0001) surface.

## Graphical abstract

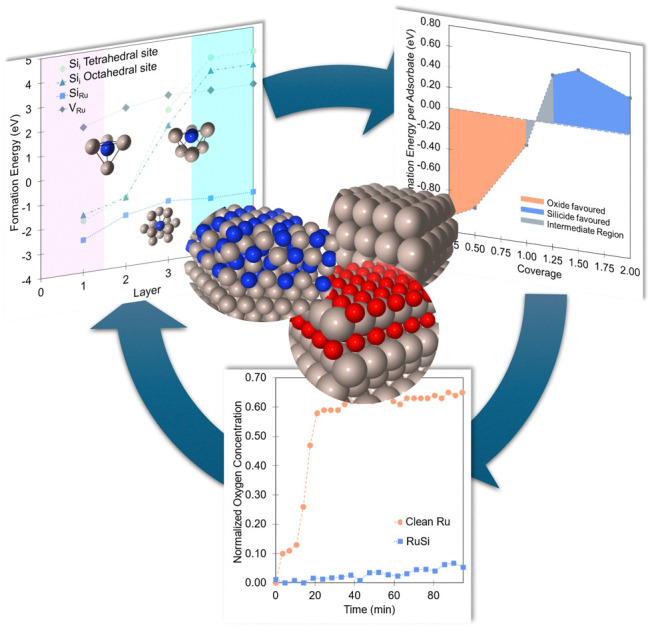

Ruthenium thin films are increasingly important for a wide variety of applications that exploit the favorable chemical, physical and electronic properties this chemical element possesses. Ruthenium has been known to have high catalytic activity originally as a homogeneous catalyst^1^ and more recently in heterogeneous catalysis.^2^ As a heterogeneous catalyst, Ru surfaces provide excellent activity for the Fischer–Tropsch process (conversion of syngas into long chain aliphatic hydrocarbons), and NH_3_ decomposition.^3–5^ Ruthenium thin films have an equally long history in a variety of protective barrier layer applications.^6–17^ Finally, combining barrier layer properties with favorable electronic properties, Ru is used as an electrode in various MOSFET, MEMs, and memory devices, where stability against oxidation is a primary concern.^18,19^

Across the highlighted use cases oxidation can be both a blessing and curse. In catalytic applications the oxidation of Ru results in a number of new active phases that show a dramatic increase in catalytic activity and scope when compared to Ru. In barrier layer and device applications, the oxidation of Ru to form RuO_2_ can be undesirable, as this goes hand in hand with the loss of the aforementioned favorable physical, chemically and electronic properties of ruthenium.^20,21^ Facilitating ion accumulation in and diffusion through the barrier layer,^21^ resulting in the modification and breakdown of the dielectric layer beneath.^22–24^ Driven by the catalysis community, the oxidation of Ru has been comprehensively studied, showing a progression from a dense hcp-monolayer to the RuO_2_ rutile structure.^2,25,26^ Theoretical models, including *ab initio* thermodynamics and lattice gas cluster expansion, align well with experimental data for the sub-monolayer coverages of Ru oxidation.^27–32^ Surface phase diagrams resulting from these models predict that the oxidation proceeds *via* a number of ordered intermediate structures.^33–37^ Guided by the previous literature characterization of Ru oxidation, we define and test a computational screening framework to assess the oxidation resistance of thin surface coatings.

Transition metal silicides have long been known to show impressive oxidation resistance in the bulk and thick film context.^15,38–43^ This oxidation resistance has been shown in many cases to extend to thin films.^40,44^ Ruthenium is known to form stable silicides with varying ratios of Si and Ru. Theoretical studies predict RuSi to be the most favored bulk composition, with the silicon-rich Ru_2_Si_3_ having a similar formation energy.^45^ Synthetically these compositions and crystal structures have been reported, using pulsed laser deposition,^46,47^ arc-melting,^48^ and various other atomic deposition approaches.^49,50^ All of the characterized compositions are semiconductors with a narrow band gap of 0.2 eV to 0.6 eV, showing an increasing band gap with increasing Si content.^45^ Studies of the Si–Ru interface show a propensity for the layers to intermix leading to the formation of Ru_*x*_Si_*y*_ interlayers, reaching thicknesses of several nanometers (≈4.7 nm).^43,51,52^

Surface deposition of Si on Ru(0001) has been studied using RAIRS, LEED, EELS, and XPS.^53,54^ With increasing coverage ordered overlayers emerge, starting from a (2 × 2) and followed by a (1.5 × 1.5) structure, corresponding to coverages of 0.25 monolayers (ML) and 0.44 ML, respectively.^53,54^ No clear characterization was possible at the higher coverages.^53,54^ For Si coverage above 0.25 ML a degree of surface mixing was reported^53,54^ and appears to accord with interface studies.^43,50–52^ However, the nature of the mixed layer is challenging to quantify and, as such, merits further investigation.

Using density functional theory (DFT) we investigate Si defects in bulk Ru to identify the possible incorporation geometries. These defects are then considered at all symmetry unique positions ranging from the bulk to the near Ru(0001) surface, and finally on-surface adsorption of Si atoms. The low energy configurations and adsorption sites for Si adatoms are subsequently used to explore the energetics of Si-layer formation as a function of coverage. These results are compared to and contrasted against the Ru_*x*_O_*y*_ system, allowing the relative stability of the forming layers to be understood and the growth modes elucidated. *In situ* XPS is used to characterize the forming silicide and verify the trend predicted by comparing the coverage dependent incorporation energies for Si and O.

All DFT simulations were performed spin-polarized at the Γ-point using the CP2K software package,^55^ employing DZVP-SR-MOLOPT basis sets^56^ and Goedecker–Teter–Hutter (GTH) pseudopotentials.^57,58^ Energy cutoffs were set to 850 Ry and 60 Ry for the relative cutoff, to give precision of 0.1 meV per atom. The pristine bulk structures were modeled as a 6 × 6 × 5 expansion of the primitive hexagonal cell and a 6 × 3 × 5 orthohexagonal cell, with both lattice vectors and ion positions relaxed (for defect and surface calculations, only the ion positions were relaxed). Atomic structures were visualized using VESTA.^59^ The surface was constructed by adding a converged vacuum slab of 20 Å along the surface normal in the *z*-direction and maintained for all structures. A 9-layers Ru surface slab was sufficient to allow the bulk defect geometries and formation energies (see Fig. 1a) to be recovered at the center of the slab (*vide infra*). The Perdew–Burke–Ernzerhof (PBE) functional,^60,61^ with D3-BJ dispersion correction^62–64^ was used for all systems, self consistent field energy set at 1 × 10^−7^ eV for energy and 0.005 eV Å^−1^ for forces.

**Fig. 1:**
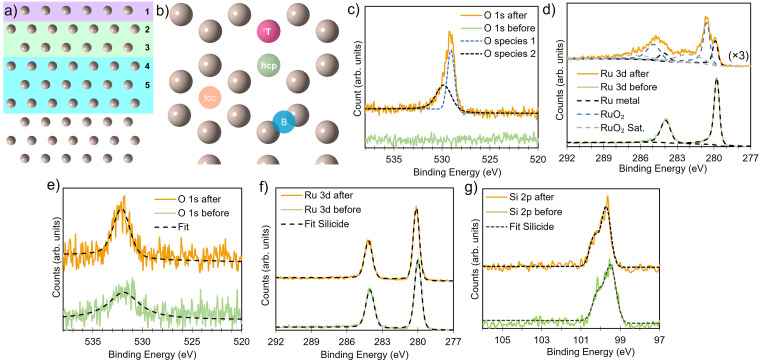
Side view (a) and top view (b) of the Ru(0001) slab, illustrating the layers of Ru considered in the defect calculations and the different types of adsorption sites. (c) and (d) Comparison of O 1s and Ru 3d XPS spectra of Ru metal before and after thermal oxidation at 340 °C and 1 × 10^−4^ mbar showing the formation of RuO_2_. For Ru silicide layers exposed to the same conditions, a small increase in the O 1s area is observed (e), whereas the Ru 3d (f), and Si 2p (g) core levels remain unchanged.

Average defect formation energies (*E*_form_) were calculated per atom according to1
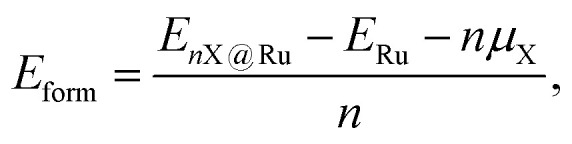
where the chemical potentials (*μ*_X_ with X ∈ {Si,O}) taken from bulk Si in the diamond structure and 
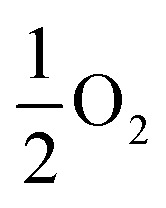
, respectively. Eqn (1) equally captures surface adsorption, where ad-atoms adsorbed on ruthenium slabs (with corresponding total energies *E*_*n*X@Ru_ and *E*_Ru_) constitute the equivalent of the bulk defects. By convention the surface coverage is referenced to hcp sites of Ru(0001) (see Fig. 1b), with one monolayer corresponding to a (1 × 1) overlayer structure with all hcp sites being occupied. Linking to structures reported in the literature, ordered overlayer structures corresponding to coverages below 1 ML are denoted by Wood's (A × B) notation commonly used in surface science.^65^ Finally, the configurational space was explored by an initial symmetry adapted enumeration of Si at the hcp and the sub-surface Si^Oct^_i_ site, based upon an expansion of the 1 × 1 hexagonal cell. The initial structures are expanded and then undergo geometry optimization, following the approach from our previous work.^66–69^ This approach produced a screening set of 252 structures with coverages ranging from (6 × 6)/0.03 ML to 2 ML. After geometry optimization the structures were further classified as mixed Ru–Si (Ru_*x*_Si_*y*_), where Ru and Si share a given layer as defined by the *z*-coordinate or layered Ru–Si (Si–Ru–Si) where Ru/Si are segregated by *z*-coordinate.

Ruthenium silicide thin films were prepared in ultra-high vacuum using pulsed laser deposition (PLD) of 30 nm thick Ru layers from a Ru target on Si(100) single crystals with a native oxide.^46,47^ After deposition in an atmosphere of 4 × 10^−2^ mbar of Ar, the samples were annealed *in situ* at 550 °C, resulting predominantly in Ru_2_Si_3_. Surface composition and oxidation state were analyzed before and after annealing and monitored *in situ* at elevated temperature in an oxygen environment. Near-ambient pressure XPS employing a Scienta Omicron HiPP-3 electron analyzer with a 1 mm entrance slit setting and a 0.8 mm cone opening was used. Oxygen dosing was controlled *via* a high-precision leak valve, and pressures were monitored using a Pfeiffer cold cathode vacuum gauge. XPS peak fitting was performed using the software KolXPD, with Shirley background and Voigt peaks for the core levels, and a Doniach–Sunjic function convoluted with a Gaussian for the metallic Ru peak (Fig. 1d).

In bulk ruthenium, Si can either be incorporated at a Ru-site (substitutional defect Si_Ru_) or at an interstitial position (interstitial defect Si_i_) as shown in Fig. 2. The lowest formation energies are −1.7 eV and 3.5 eV, respectively, with the Si_Ru_ being substantially more favored. The substitutional Si_Ru_ defect is energetically favored with Si readily accommodated at the Ru lattice site with negligible distortion. The coordination is maintained with a 0.03 Å deviation with respect to the defect free Ru-site. A charge transfer from Ru to Si results in a Mulliken charge of *q*_Mul_ = −0.20, predominantly *via* donation from the first coordination shell.

**Fig. 2:**
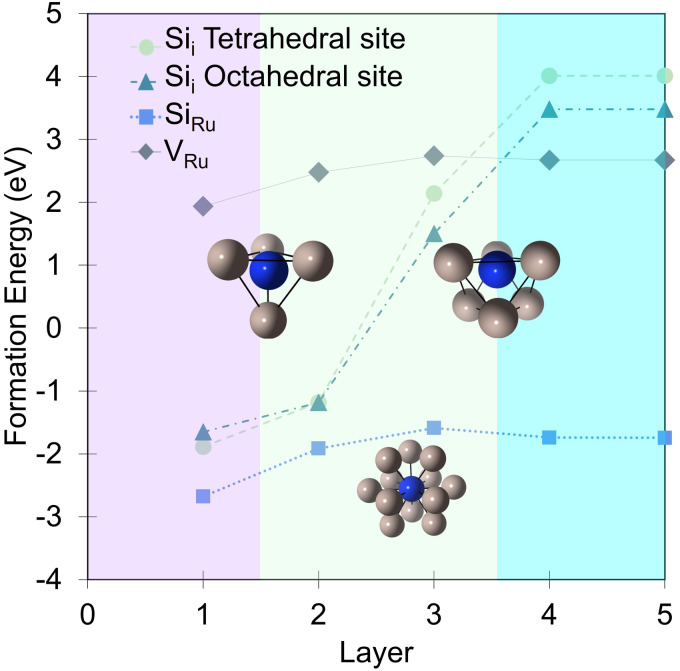
The associated formation energies for the Si_Ru_, Si_i_, and V_Ru_ defects in each of the indicated layers (Fig. 1a). Purple shaded area indicates surface layer, green sub-surface, and blue bulk. Grey spheres are Ru and dark blue Si.

There are two Si_i_ sites: octahedral (*E*_form_(Si^Oct^_i_) = 3.5 eV) or tetrahedral (*E*_form_(Si^Tet^_i_) = 4.0 eV). The distortion induced by the incorporation of the large Si atom results in a significant relaxation in the surrounding Ru extending out to the next-neighbor shell driving the significant *E*_form_ resulting in a Si_i_ bulk defect concentration of effectively zero under equilibrium conditions. For Si^Oct^_i_ the octahedral symmetry is distorted by a shift of the Si-atom along the [0001] crystallographic direction resulting in 3 × 2.10 Å and 3 × 2.35 Å Ru–Si separations. The steric crowding is more pronounced for Si^Tet^_i_ (3 × 2.16 Å and 1 × 2.09(9) Å), driving a more dramatic distortion, as expressed in the less favorable *E*_form_. In both cases, and in contrast to Si_Ru_ charge transfer is from the Si to the neighboring Ru in the first coordination shell resulting in Mulliken charges of *q*_Mul_(Si^Oct^_i_) = +0.31 and *q*_Mul_(Si^Tet^_i_) = +0.24, accompanied by compensating negative charges on the six and four neighboring Ru, respectively.

To evaluate the energetic impact of defects transitioning from the bulk to the Ru(0001) surface, the formation energies of a ruthenium vacancy (V_Ru_), Si_i_ (in tetrahedral and octahedral symmetry sites) and Si_Ru_ defects were calculated within the first five layers (Fig. 2). Defects placed in the fifth layer recover the bulk defect formation energy and geometry. Interestingly and perhaps not unexpectedly the defects are more energetically favorable at the surface when compared to the bulk. The Si_Ru_ defect exhibits a smaller energetic preference for the surface, being 0.8 eV more favorable, underscoring its ease of incorporation. The V_Ru_ shows a similar trend albeit from a significantly higher formation energy that the Si_Ru_. In contrast, the Si_i_ defect shows substantial energy reductions of 1.87 eV and 1.98 eV for the tetrahedral and octahedral sites, respectively, from the fifth to the third layer. The energy difference of approximately 0.5 eV between these interstitial sites is maintained until the second layer, where the defects become energetically equivalent due to isostructural relaxation, resulting in the displacement of a Ru atom to form a Ru_ad_ adatom and accommodate Si_Ru_ at the surface. At the surface layer, both Si^Oct^_i_ and Si^Tet^_i_ relax to form Si adatoms (Si_ad_) at fcc and hcp sites, respectively. Interstitial Ru defects (Ru_i_) were examined, but were found to have a formation energy of 8.35 eV in the bulk so was not considered in the surface study. Similar to other well-studied adsorbates like O^28,30,32,70^ and N^70–72^ on Ru(0001), the hcp site is the lowest energy adsorption site, with the fcc site being 0.32 eV higher in energy.

In agreement with previous experimental studies^53,54^ Si uptake upon prolonged exposure progresses *via* a number of ordered surface adsorption phases, with the (2 × 2) being most favored at low Si-coverages (Fig. 3a, blue circle at 0.25 ML). Focusing on the energetically most favorable structures shown in Fig. 3a, taken separately for the two different modes of adsorption at each coverage, results in Fig. 3b. The forming Si-layer shows a propensity for mixing and at a Si-coverage of 0.5 ML the mixed layer becomes isoenergetic with the surface adsorbed layer (Fig. 3b). As the Si concentration increases the mixed phases become dramatically more favored for coverages up to 1 ML, which represents a mixed stoichiometric-RuSi layer. Above 1 ML, the *E*_form_ shows a negligible decrease as the Si concentration increases to 2 ML. Interestingly, the work function (WF) is broadly insensitive to Si coverage (Fig. 3c) with the Si–Ru–Si layer showing only a small variation and no meaningful change in WF compared to the clean Ru(0001) (4.92 eV), to 2 ML Si coverage (4.90 eV). The shift is more pronounced for the mixed layers with a WF of 5.14 eV at 2 ML, which is already approaching that of RuSi (5.2 eV). In both cases, the WF shift is far less dramatic than reported (2.5 eV) for the oxidation of Ru(0001).^28^ A similar trend is seen in the DoS with extensive hybridisation between the Ru-d and the Si-p states which is apparent from the single Si-adsorption forward. The surface states are initially predominately Si-character (Fig. 4a) becoming increasingly mixed character (Fig. 4b and c).

**Fig. 3:**
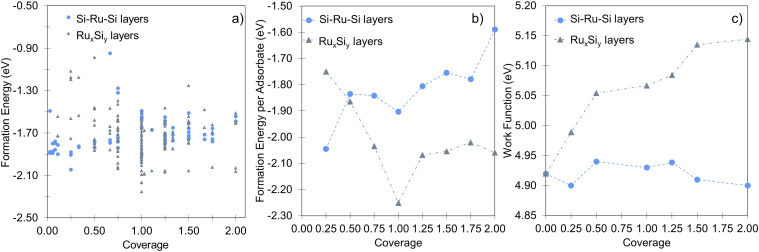
(a) The complete sampling of the Ru_*x*_Si_*y*_ representing 252 structures, encompassing all of the structures when broken down by type gives the trend shown in (b). (b) The formation energy comparison of the mixed (Ru_*x*_Si_*y*_ – grey triangles) and segregated (Si–Ru–Si – blue circles) layers showing the layered structures are favored at and below 0.5 ML, whereas the mixed structures become increasingly favored above 0.5 ML. (c) Illustrates the evolution of the work function for both the layered and the mixed structures, both sit in a narrow range while showing the opposite trend in coverage dependence.

**Fig. 4:**
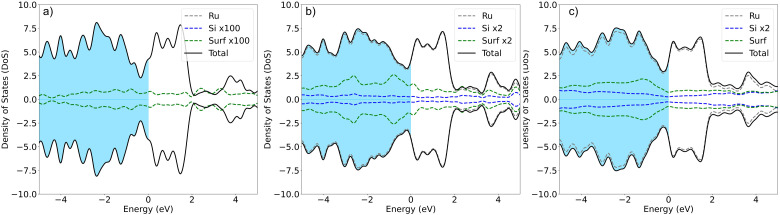
The DoS for (a) a single Si at the hcp site, (b) 1 ML mixed layer coverage, and finally (c) 2 ML coverage. For clarity in each case the Si-peak has been magnified, the scaling factor is highlighted in the legend, in each case Ru is grey, Si is blue and the surface density of states from the forming Ru–Si is green, the blue coloured region shows the occupied states and by convention the Fermi level is at 0 eV.

In that vein, it is instructive to compare the exposure of Ru(0001) to silicon with oxygen in more detail based on energetics that are important for the interface and overlayer formation. Oxygen structures were sampled from 0 to 2 ML, adopting the same methodology used for Si. These results accord well with the previous studies of Reuter and co-workers.^27–29^Fig. 5a illustrates the formation energy per Si and O atom across varying coverages, while Fig. 5b shows the relative energies for Ru_*x*_Si_*y*_ and Ru_*x*_O_*y*_ as a function of coverage. Notably, no stable mixed Ru–O configurations are found. Instead, oxide growth follows a layered structure, favoring the O_i_ configuration over O_Ru_ + Ru_ad_. Below 1 ML, O-layer formation is significantly favored, decreasing in stability by +0.6 eV per atom up to 1 ML. Above 1 ML, the Ru_*x*_Si_*y*_ and Ru_*x*_O_*y*_ structure become isoenergetic and above 1.25 ML, Ru_*x*_Si_*y*_ is favored (Fig. 5b). The *E*_form_ for Ru_*x*_Si_*y*_ structures is largely independent of coverage, highlighting the limited interaction between neighboring Si as described for the single atom. For Ru_*x*_O_*y*_, the opposite is true, with a significant lateral interaction (≈+0.35 eV). This interaction is comprised of both Coulombic repulsion between neighboring oxygen anions and significant lateral strain induced by local relaxation. Secondly, the negligible Si–Si interaction and the propensity to form mixed layers stabilize the forming silicide, resulting in a reduction Δ*E*_form_ = −0.2 eV per atom as shown in Fig. 3a, whereas the O-layer shows an increase in Δ*E*_form_ = +1.75 eV per atom over the same coverage range (Fig. 5a). The result is shown in Fig. 5b, below 1.0 ML the O-layer is favored above 1.25 ML Ru_*x*_Si_*y*_ is favored.

**Fig. 5:**
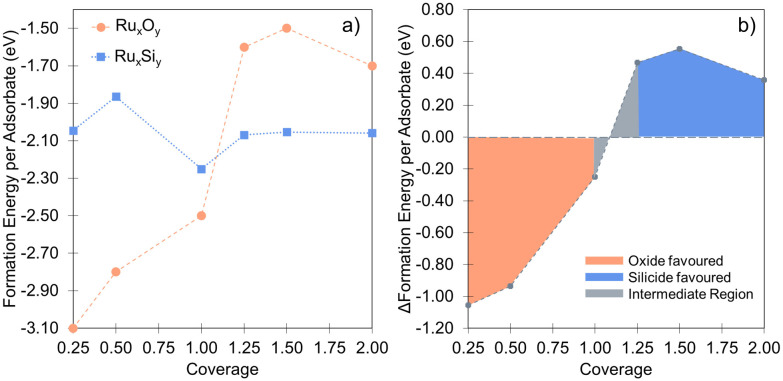
(a) Comparison between the coverage dependence of the formation energies for Si and O adlayers. For each coverage, the Si points are the lowest energy configurations from Fig. 3a. The difference of formation energies between Si and O adlayers are plotted in (b), visualizing the coverage dependent stability of the two systems with respect to each other.

It is important to note that the energetic treatment presented here neglects the kinetics inherent in the process, which may play an important role. Hence, this approach should only be used as an initial screen with further theoretical or experimental investigation required to confirm the prediction. To verify whether the growth kinetics inherent in the process play an important role, the predicted stability of Ru_*x*_Si_*y*_ with respect to RuO_2_ formation is investigated experimentally by means of *in situ* XPS (Fig. 6a). The response of polycrystalline Ru and Ru_*x*_Si_*y*_ layers upon annealing in an oxidizing atmosphere is measured. Fig. 6 shows the variation in oxygen content over time at 340 °C under 1 × 10^−4^ mbar of O_2_. For polycrystalline Ru, rapid oxidation is observed, reaching a saturation level slightly above 60% (Fig. 6b). This is indicative of the formation of a RuO_2_ overlayer with a thickness of several nanometers. In contrast, under the same conditions, the RuSi layer exhibits remarkable stability, with only a marginal increase in oxygen content (≈5%) after an hour of annealing. Based on the unchanged peak positions and peak shapes of the Si 2p and Ru 3d core level spectra, this minimal change is interpreted as surface decoration rather than substantive oxidation. Hence, providing confirmation of the oxidation stability of Ru_*x*_Si_*y*_ predicted by DFT calculations extends to the macroscale. Furthermore, the lack of subsurface oxygen accords with the energetic trends predicted from our DFT calculations, and aligns with previously reported EELS and SIMS measurements where O was only found at the Ru_*x*_Si_*y*_ surface.^50^

**Fig. 6:**
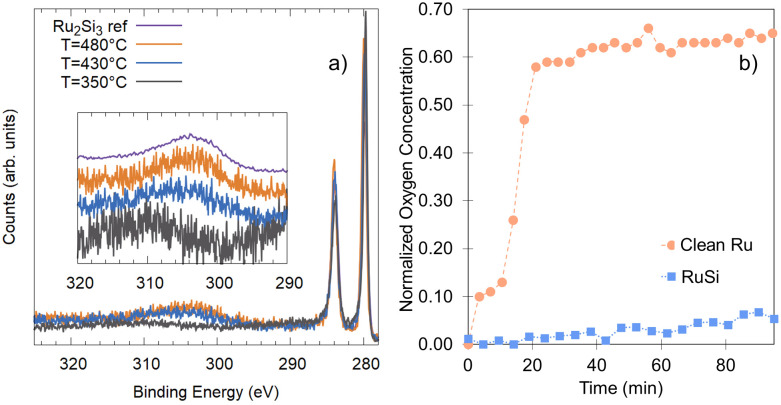
(a) Ru 3d XPS characterization of the silicide formation as a function of temperature, performed *in situ* during heating and referenced to Ru_2_Si_3_ previously described.^46^ (b) Comparison of the oxygen uptake of a clean Ru-surface (orange circles) and a Ru_*x*_Si_*y*_-surface (blue squares) at 340 °C in 1 × 10^−4^ mbar of O_2_ based on the relative oxygen content measured by XPS.

Employing DFT, the energetic drivers for the formation of Ru_*x*_Si_*y*_ films were described, and the structure-coverage relationship elucidated. Our analysis revealed the facile mixing behavior between Ru and Si, which becomes increasingly energetically favored above 0.5 ML, driving the formation of the Ru_*x*_Si_*y*_ observed by *in situ* XPS. The suitability of Ru_*x*_Si_*y*_ layers to act as a protective layer for Ru was investigated by comparing the relative stabilities of the oxide and silicide. Importantly, we demonstrated that for coverages above 1 ML, silicide formation becomes increasingly energetically favored with respect to the oxide. The postulated effectiveness of Ru_*x*_Si_*y*_ in preventing the formation of RuO_2_ has also been verified by *in situ* XPS measurements. Together, the DFT calculations and XPS measurements provide a comprehensive validation of Ru_*x*_Si_*y*_'s oxidation stability, bridging atomic-scale stability predictions with macroscale experimental confirmation under near-ambient conditions. Furthermore, the methodologies applied here offer a convenient framework for the initial screening of surface layer formation and associated stability prior to more detailed computational characterisation and experimental testing. The insights gained from this study could guide the design of more robust and oxidation-resistant Ru-based materials for applications where long-term stability is paramount.

## Author contributions

JC: conceptualisation, formal analysis, investigation, methodology, validation, visualisation, writing – original draft preparation. SvV: formal analysis, investigation, validation, visualisation, writing – review & editing. JM: funding acquisition, project administration, resources, supervision, writing – review & editing. RB: funding acquisition, project administration, resources, supervision, writing – review & editing. EO: conceptualisation, funding acquisition, investigation, methodology, project administration, resources, supervision, writing – original draft preparation, writing – review & editing.

## Data availability

The datasets generated during the current study are publicly available *via* a Zenodo repository at https://doi.org/10.5281/zenodo.12604806.

## Conflicts of interest

There are no conflicts to declare.
